# Cardiovascular Remodeling and Potential Controversies in Master Endurance Athletes—A Narrative Review

**DOI:** 10.3390/life15071095

**Published:** 2025-07-12

**Authors:** Othmar Moser, Stefan J. Schunk, Volker Schöffl, Janis Schierbauer, Paul Zimmermann

**Affiliations:** 1 Division of Exercise Physiology and Metabolism, BaySpo-Bayreuth Center of Sport Science, University of Bayreuth, 95447 Bayreuth, Germanyjanis.schierbauer@uni-bayreuth.de (J.S.); 2 Exercise Physiology, Training & Training Therapy Research Group, Institute of Human Movement Science, Sport and Health, University of Graz, 8010 Graz, Austria; 3 Interdisciplinary Metabolic Medicine Trials Unit, Department of Internal Medicine, Division of Endocrinology and Diabetology, Medical University of Graz, 8036 Graz, Austria; 4 Department of Internal Medicine IV, Nephrology and Hypertension, Saarland University, 66421 Homburg/Saar, Germany; schunkstefan@gmx.de; 5 Department of Nephrology, Hypertension and Rheumatic Disease, Klinikum Bamberg, 66424 Bamberg, Germany; 6 Interdisciplinary Center of Sportsmedicine Bamberg, Klinikum Bamberg, 96049 Bamberg, Germany; volker.schoeffl@me.com; 7 Department of Traumatology and Orthopaedics, Klinikum Bamberg, 96049 Bamberg, Germany; 8 Department of Trauma Surgery, Friedrich Alexander Universität Erlangen-Nürnberg, 91054 Erlangen, Germany; 9 Section of Wilderness Medicine, Department of Emergency Medicine, University of Colorado School of Medicine, Aurora, CO 80045, USA; 10 School of Health, Leeds Beckett University, Leeds LS1 3HE, UK; 11 Department of Cardiology, Klinikum Bamberg, 96049 Bamberg, Germany

**Keywords:** master athlete, cardiovascular risk, atrial fibrillation, coronary artery atherosclerosis, cardiac remodeling, nutritional supplements

## Abstract

While the interest and participation in general endurance training and recreational sports competitions have continuously increased in recent decades, the number of recreational master-level endurance athletes has additionally multiplied. Athletes, active men and women older than 40 years of age, who participate in competitive athletics are usually referred to by the term master athletes (MAs). Previous research revealed the significant benefits of regular moderate physical activity, i.e., its positive influence on cardiovascular risk factors and cardiovascular health; however, recent data have raised concerns that long-term endurance exercise participation is associated with cardiac remodeling and potential adverse cardiovascular outcomes. Previous research also indicated potential structural, functional, and electrical remodeling in MAs due to prolonged and repeated exposure to high-intensity endurance exercise—a condition known as athlete’s heart. In this review, we focus on the association between extreme levels of endurance exercise and potential cardiovascular controversies, such as arrhythmogenesis due to new-onset atrial fibrillation, accelerated coronary artery atherosclerosis, and exercise-induced cardiac remodeling. Additionally, the exercise-dependent modulation of immunological response, such as proteomic response and cytokine alterations, is discussed. Furthermore, we discuss the impact of nutritional supplements in MAs and their potential benefits and harmful interactions. We aim to provide sports medicine practitioners with knowledge of these contemporary longevity controversies in sports cardiology and to highlight the importance of shared decision making in situations of clinical uncertainty.

## 1. Introduction

While the interest and participation in general endurance training and recreational sports competitions has continuously increased in recent decades, the number of recreational master-level endurance athletes has additionally multiplied [1]. In this context, master athletes (MAs) are men and women, generally older than 40 years of age, who participate in competitive athletics [1].

Previous research revealed the positive effects of regular and moderate physical activity on cardiovascular health; however, recent scientific studies have shown an increase in long-term adverse cardiovascular outcomes due to regular ambitious endurance sport activity [1,2,3,4]. The current general guidelines for regular physical activity, in which just 150 min of moderate anaerobic exercise or 75 min of vigorous-intensity physical activity per week is recommended, are associated with substantial health benefits [1,5]. MAs usually exceed these thresholds, triggering wide debate on the potential negative effects of vigorous endurance exercise training [1,5,6]. In this context, continuous lifelong endurance sport participation, especially high-level training and the lifelong accumulation of training volumes, is considered to result in cardiac structural, functional, and electrical remodeling in MAs—a condition referred to as athlete’s heart. The important factors that determine exercise-induced cardiac remodeling (EICR) are the type, frequency, and duration of exercise and the lifetime dose of exercise exposure, as well as nutritional habits, age, sex, ethnicity, and the presence of relevant comorbidities [1,7,8]. Various phenotypes of athlete’s heart have been revealed, and high-intensity endurance exercise might serve as a potential trigger for negative cardiovascular remodeling in MAs, such as an elevated risk of arrhythmogenesis [2,3,4,9,10,11].

Various negative consequences and controversies regarding EICR have been discussed, and previous research has revealed the foremost association between an increased risk of premature onset of paroxysmal atrial fibrillation (AF) in MAs and high-level endurance training [1,11,12,13,14]. Additionally, left atrial (LA) dilatation and subsequent fibrosis; chronic periodic inflammation and fluctuations in circulating protein levels, such as those of interleukin-6, C-reactive protein, and insulin-like growth factor-1; vagal nerve activity in endurance athletes; enhanced sympathetic nervous system activity during ambitious exercise; variable alcohol consumption; and psychosocial stress appear to increase the risk of paroxysmal AF, especially in male MAs [1,9,14,15,16,17].

The topic of endurance sports activity and increased arteriosclerosis in MAs is controversial whilst being of great interest from a pathophysiological point of view. Previous research revealed increased coronary artery calcification (CAC) in MAs; at the same time, it could not highlight a more favorable coronary plaque composition due to a healthy lifestyle and ambitious endurance sport participation [1,10]. Therefore, lifelong middle-aged MAs showed a higher overall plaque burden—in particular, more non-calcified and mixed plaques and more potentially hemodynamically relevant plaques in proximal segments—compared to healthy individuals with an equivalent cardiovascular risk profile [10]. Based on these observations, the dose–response relationship between CAC and endurance training appears to follow an inverted J-shaped curve [10].

However, MAs are generally considered to be healthier than their sedentary peers. While chronic conditions become more prevalent in MAs, pharmacological and non-pharmacological interventions, such as the use of nutritional supplements (NSs), are of broad interest in this cohort [18]. In comparison to young elite athletes, who use NSs to enhance their athletic performance, MAs predominantly use supplements for health reasons and the maintenance of individual performance. Stiegel et al. reported that up to 60.5% of MAs use NSs [19], and the most prevalent supplements are dietary supplements (DSs), such as vitamins (55.8%), calcium supplements (23.1%), glucosamine and chondroitin (11.6%), coenzyme Q10 (CoQ_10_; 5.4%), and caffeine [18].

In addition to the EICR mentioned above, our review aims to highlight the potential beneficial aspects of NSs in MAs and to discuss their potential interactions, for which the current scientific literature and available evidence are scare.

## 2. Materials and Methods

For this narrative review, a non-systematic literature search was performed in the PubMed database between January 2025 and June 2025, searching for studies, case reports, and review articles in the context of cardiovascular remodeling and potential cardiovascular controversies, along with the relevant nutritional and dietary supplements widely used by MAs. Key articles from these topics of research were included based on the writing group’s decision.

## 3. Structural, Functional, and Electrical Cardiac Remodeling—Specific Athlete Risk

**Figure 1:**
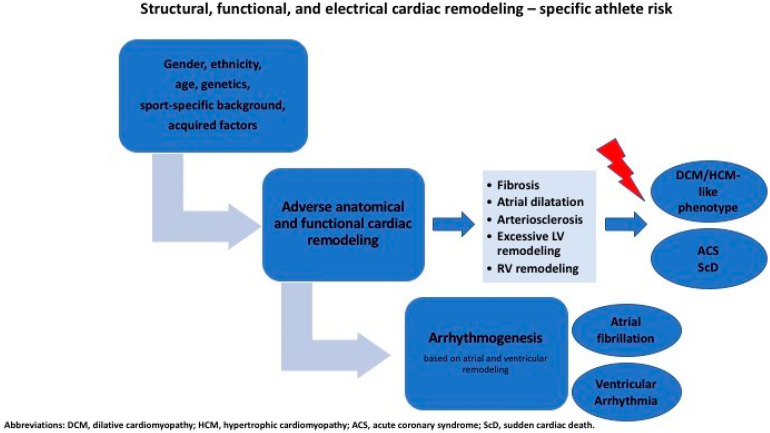
Structural, functional, and electrical cardiac remodeling—specific athlete risk. Abbreviations: DCM, dilative cardiomyopathy; HCM, hypertrophic cardiomyopathy; ACS, acute coronary syndrome; ScD, sudden cardiac death.

The potential negative consequences and controversies regarding structural, functional, and electrical cardiac remodeling determining a specific athlete’s risk, i.e., arrhythmogenesis, cardiac fibrosis, and accelerated atherosclerosis, are discussed in detail in the following section, as displayed in Figure 1.

### 3.1. Association Between Endurance Training and Atrial Fibrillation

AF is the most frequent arrhythmia in general clinical practice and is associated with increased risks of heart failure, stroke, peripheral embolism, and mortality [20,21]. Current data reveal the global burden of disease to be 33.5 million patients [20,21]. Therefore, even in the general population, great efforts have been made to identify modifiable risk factors to establish preventive strategies, thereby minimizing the burden of AF [20,21]. In this context, electrical and structural remodeling of the atrial tissue due to different pathogenic mechanisms and various influencing circumstances, such as dietary habits, potential genetic burden, and sedentary lifestyle, play a significant role in the management of AF [20,21,22].

Additionally, previous research in sports cardiology revealed an increased risk of early-onset AF in endurance MAs [1,11,12,13,14]. Nevertheless, the association between early-onset AF and long-term endurance training may represent the foremost associated issue for MAs in the context of potential negative long-term cardiovascular controversies. Previous research demonstrated an increased risk of premature-onset paroxysmal AF in MAs and those participating in high-level endurance training due to EICR, i.e., potentially negative anatomical and functional cardiac remodeling [1,11,12,13,14]. High-intensity training and the accumulation of lifetime training hours during an athlete’s career seem to predispose them to an increased risk of early onset, notably PAFIYAMA syndrome (paroxysmal AF in young and middle-aged athletes), especially in male endurance athletes [2,11,13,14,23,24,25].

Johansen et al. revealed a high prevalence of AF in older male endurance MAs, with a risk ratio (RR) of 1.88 compared with non-athletes [26]. Additionally, Abdulla et al. and Andersen et al. were able to elucidate significant higher risks of AF and arrhythmogenesis in male athletes participating in vigorous endurance sports [27,28]. In summary, a J-shaped association between physical activity and the incidence of AF can be observed, such that moderate and excessive physical activity may affect AF’s incidence based on different pathophysiological mechanisms [28,29]. The prevalence of AF, which has been studied in selected studies on MAs, is displayed in Table 1.

The pathogenesis of AF in MAs seems to be multifactorial and complex. Next to anatomical and functional EICR, the following factors and potential interactions appear to increase the risk of paroxysmal AF, especially in male MAs. The accumulating prevalence of AF in male endurance athletes appears to be associated with chronic periodic inflammation status, vagal nerve activity, enhanced or even overwhelming sympathetic nervous system activity during ambitious exercise, variable alcohol consumption, and psychosocial stress levels [1,9,14,15,16]. Peritz et al. were able to characterize and quantify fibrotic changes within the LA in highly trained endurance athletes, which might represent an early indicator for enhanced risk of EICR and subsequent arrhythmogenesis [25]. In this context, the exercise-dependent modulation of the immunological response pathway in endurance MAs must be emphasized [17]. Alterations in the proteomic response due to endurance exercise, involving immune modulation (i.e., growth/differentiation factor 15), cell death (i.e., histones), inflammation (i.e., interleukin-6 levels), and skeletal muscle metabolism (i.e., α-actinin-2), seem to play an important role in AF prevalence in endurance MAs [17]. Local atrial fibrosis, displaying a profibrotic substrate in exercise-related AF, is significantly associated with increased levels of fibrotic markers, i.e., fibronectin-1 and matrix metalloproteinase type I, in animal models [17,30].

### 3.2. Accelerated Coronary Artery Atherosclerosis in Endurance MAs

Next to AF, accelerated CAC is one of the main topics to be addressed in endurance MAs. Atherosclerotic cardiovascular disease (ASCVD) and subsequent major adverse cardiac events (MACEs) are the most common cause of death in western industrialized nations [31]. ASCVD is a multifactorial dynamic process that leads to CAC with different plaque morphologies. Cardio CT is used to further differentiate CAC, to analyze its extent and plaque morphology based on the Agatston units (AU), and to perform appropriate risk stratification in cardiovascular patients [1].

Previous research has demonstrated that regular, moderate physical activity confers significant benefits on cardiovascular health, lowering the risk of ASCVD compared to a sedentary lifestyle [1,2,3,4,32,33]. Contrary to this established view, several studies have demonstrated a paradoxical dose–response relationship between coronary artery disease (CAD) and endurance training, which might be inverted J-shaped [23]. Previous data from Mohlenkamp et al. and Schwartz et al. demonstrated increased CAC volumes in male marathon runners compared to matched non-athletic controls [34,35]. Although there seems to be an association between intensive endurance training and CAC, the plaque morphology in athletes seems to be favorable: their coronary plaques are often more calcified, indicating a more stable plaque composition with lower rupture risk [3,36]. In this context, Aengevaeren et al., 2017 and Merghani 2017 reported increased overall CAC and a higher prevalence of atherosclerotic plaques in highly trained male MAs with >2000 MET (metabolic equivalent of task) minutes per week compared to men with lower activity levels or sedentary lifestyles [3,36]. Increased cardiovascular fitness is independently associated with a reduced risk of MACEs. Notably, DeFina et al. found no increase in mortality among high-endurance MAs with >3000 MET min per week and CAC scores greater than 100 AU over a 10-year follow-up period [37].

Nevertheless, the underlying pathophysiological mechanisms driving the accelerated progression of CAC in male MAs remain unclear. Several influencing factors have been discussed. Earlier research revealed evidence of increased shear stress on the coronary arteries during repetitive endurance exercise, determining CAC [38]. Therefore, the tendency toward calcified plaques in endurance MAs might be an adaptive phenomenon with a lower risk of plaque rupture and associated MACEs [39]. Additionally, earlier research by Zerath et al. indicated enhanced exercise-related release of parathyroid hormone (PTH) and total calcium accompanied by reduced osteocalcin levels with endurance training in elderly participants [40]. Nevertheless, high-intensity endurance training tends to increase the systemic inflammation status. In particular, marathon running results in enhanced plasma interleukin-6 and interleukin-8 levels and neutrophil recruitment that might be associated with tissue damage [41]. Therefore, the question arises as to whether the potential imbalance of pro- versus anti-inflammatory agents in endurance MAs might contribute to vascular endothelial damage and CAC progression [41]. Nevertheless, the current scientific data cannot definitively clarify this topic.

Current scientific data predominantly focus on male endurance MAs, so evaluating the gender-specific risk of physical activity and CAC progression remains an important future research direction [1].

### 3.3. Exercise-Induced Cardiac Remodeling, Cardiac Fibrosis, and Arrhythmogenesis in Endurance MAs

In addition to the two phenomena described above, namely, arrhythmogenesis and accelerated CAC in endurance MAs, functional–architectural remodeling in terms of myocardial fibrosis (MF) represents a clinically relevant topic in athletes. It is unclear whether MF represents a consequence of the systemic inflammatory response or localized mechanical stimulation [17].

MF is a common finding in diverse cardiac diseases and is associated with a high prevalence of sudden cardiac death (SCD) [42]. While regular physical activity is known to be beneficial for cardiovascular health, higher doses of vigorous endurance training have been reported to be associated with myocardial fibrosis and EICR [1,8,42]. These EICR changes in endurance MAs depend on numerous parameters, and earlier research revealed large inter-individual variability when it comes to the sport discipline, the duration and lifetime dose of exercise exposure, and the athlete’s gender and ethnicity [1,8]. While LA fibrosis and structural and functional LA remodeling are known phenomena in highly trained endurance MAs (especially male MAs) [14,25], left ventricular (LV) chamber enlargement due to cardiac preload and afterload is associated with a 10–20% increase in wall thickness and 10–15% cavity enlargement [13]. Consequently, in daily clinical practice, it is challenging to differentiate between EICR and pathological early-stage cardiomyopathy. Therefore, physiological sport-specific remodeling should always be evaluated continuously and against the ethnic and sport-specific background in MAs.

In this context, Breuckmann et al. detected high rates of late gadolinium enhancement (LGE) in male marathon runners compared to non-active controls [43]. Other observational studies revealed a certain prevalence of MF even in the general population [44,45]. The evidence to date of MF in athletic populations is based on observational data and does not provide a sufficient explanation for the underlying mechanisms [42]. Firstly, genetic predisposition to cardiomyopathies might contribute to the development of MF during a person’s lifetime and its variable presentation in MAs. Genetic alterations in sarcomere proteins, calcium-handling proteins, or Z-disks predisposingly encode for hypertrophic cardiomyopathy (HCM)—a frequent cause of SCD in athletes due to LGE in the LV and associated malignant ventricular arrhythmogenesis [46]. Arrhythmogenic right ventricular cardiomyopathy (ARVC) represents yet another genetic cardiomyopathy and is characterized by pathologic fibrofatty replacement, mainly of the RV [47,48,49]. It is predominantly based on a genetic disorder in mutations encoding for desmosomes. Previous research provided some evidence of malignant ventricular arrhythmogenesis and SCD due to frequent endurance exercise and supported a recommendation of exercise restriction in ARVC patients and male MAs [47,48,49]. Secondly, continued exercise training in endurance MAs in subclinical viral myocarditis might contribute to chronic inflammation and LGE development [50]. Additionally, high training volumes in endurance training are associated with transient exercise-induced changes in the RV and LV’s anatomic and functional structure, from which a full recovery within days has been reported [42,51]. Therefore, focal LGE has been reported in the septum and RV insertion points in 48% of athletes [42]. These findings might be due to chronic mechanical stress resulting from prolonged exercise and exercise-associated pulmonary artery pressure overload with subsequent elevated pulmonary artery pressure [42,52]. The repetitive increase in pulmonary pressure without a compensatory decrease in pulmonary vascular resistance might contribute to RV maladaption, i.e., pathological RV remodeling, scarring, and enhanced arrhythmogenetic substrates [53]. Other studies revealed increased cardiac troponin I and T levels after prolonged exercise [54], presumably displaying high-intensity endurance exercise-induced cardiac microdamage associated with MF development after lifelong exercise training [42,55]. Eijsvogels et al. revealed exercise- and intensity-related elevated troponin levels in 100% of Boston marathon finishers [56,57]. These findings support the evidence of cardiac fatigue, defined as transient myocardial dysfunction, due to intense endurance training [58].

Nevertheless, the prognostic significance of non-specific MF patterns in athletes remains uncertain [42]. On the one hand, previous studies demonstrated a higher prevalence of coronary events and higher CAC to be associated with MF in runners [35,59], whereas other studies did not support the thesis of arteriosclerosis and previous infarction as the cause of LGE [42]. At present, there is no evidence that MAs with a non-specific MF pattern should be restricted from exercise. Incidental MF findings should be evaluated cautiously in an athlete’s care and judged with regard to the athlete’s clinical presentation [42].

## 4. Use of Medication and Dietary Supplements in MAs—Potential Interactions

In addition to the described potential cardiovascular controversies due to EICR in MAs, NSs and their potential interactions must be a focus in the sports medicine care of aging athletes.

**Figure 2:**
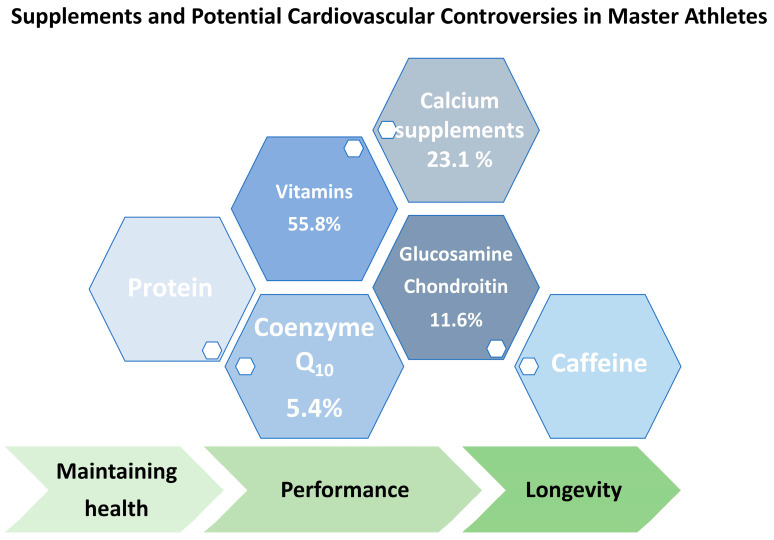
Supplements and potential cardiovascular controversies in MAs.

MAs are generally considered to be healthier than their sedentary peers. Despite the limited scientific research on chronic diseases and conditions in MAs, it seems to be evident that MAs are less associated with cardiovascular, metabolic, and hormonal diseases, including hypertension, hyperlipidemia, all types of cancer combined, osteoporosis, arthritis, anxiety, and depression [18,60,61]. However, chronic conditions become more prevalent in any population with age; therefore, pharmacological and non-pharmacological interventions, such as the use of NSs among MA patients, are a key topic of focus [60]. Unlike elite athletes, who use NSs to enhance their athletic performance, MAs predominantly use these substances for health reasons and, therefore, have closer contact with the healthcare system [19]. Stiegel et al. reported that 60.5% of MA patients use NSs [19]. The substances used most frequently are vitamins (35.4%) and minerals (29.9%). Guthrie et al. reported similar findings in U.S. Masters Swimming (USMS) members, among whom the overall prevalence of supplement use was 62% [18,62]. The most prevalent supplements were DSs, such as vitamins (55.8%), calcium supplements (23.1%), glucosamine and chondroitin (11.6%), coenzyme Q10 (CoQ_10_; 5.4%), and caffeine [18], as displayed in Figure 2. Recent evidence indicates potential benefits for MAs to maintain performance and health via the intake of protein (potentially via supplements), creatine (e.g., via supplements), or nitrate (e.g., via beetroot juice) [18,63,64,65,66].

The coincidence of athletes’ awareness of maintaining health and longevity using DSs on the one hand and the increased risk of cardiovascular events and chronic conditions with increasing age on the other hand might raise the possibility of potential adverse interactions with accompanying medication [18]. These potential interactions might occur due to an alteration in pharmacodynamic or pharmacokinetic mechanisms [67]. In our review, we would like to focus on glucosamine and chondroitin, coenzyme Q_10_ (CoQ_10_), red yeast rice extract, calcium supplements, creatine, and caffeine and their potential beneficial and harmful effects and interactions in MAs.

Glucosamine and chondroitin, commonly used by MAs for osteoarthritis pain and disability, are thought to interact with warfarin taken concurrently, as reported in previous case reports [68,69,70]. Warfarin, as an anticoagulant medication commonly used in patients with prosthetic heart valves and AF, might be negatively affected by glucosamine and chondroitin intake via the potentiation of warfarin’s effect and an increase in bleeding risk [68,69,70].

CoQ_10_, known and commonly used for its antioxidant and mitochondrial bioenergetic properties, is used substantially more often by MAs than by the general population [62,71]. Potential drug interactions between CoQ_10_ and vitamin K might occur due to their structural similarity, with a reduction in warfarin’s anticoagulative effects [71]. Another potentially relevant interaction in MAs suffering from CAC might be with statin therapy for hyperlipidemia and CAC. Statins lower CoQ_10_ levels by inhibiting the enzyme 3-hydro-3-methylglutaryl Coenzyme-A (HMG-CoA) reductase and might thereby mediate a statin-induced CoQ_10_ deficiency and subsequent statin-associated muscle symptoms [72]. Additionally, CoQ_10_ has been reported to have hypotensive and antioxidant effects; therefore, monitoring blood pressure and blood glucose levels may be indicated to accompany the use of antihypertensive and antidiabetic medication in MAs [73,74].

Red yeast rice extract has been reported to have cholesterol-lowering properties and is therefore commonly used in Asia and Europe, even by MAs, as an additional or single lipid-lowering medication [18,62,72]. Previous research identified red rice extracts containing lovastatin-like compounds that inhibit several drug-metabolizing cytochrome P450 enzymes and drug-transporter P-glycoprotein activity and, therefore, might be responsible for potential medication interactions, especially with statins, antibiotics, or psychopharmaceuticals [18,75,76].

Calcium, a common supplement among MAs, is known for its potential interactions with bisphosphonates and some antibiotics [77,78]. Therefore, the common use of calcium, bisphosphonates, and antacid medication might have negative effects on the bioavailability of oral bisphosphonates in the general population and even in MAs [77,78]. Furthermore, the co-administration of calcium might negatively affect antibiotic treatments, especially those with quinolones and tetracyclines, by reducing their gastrointestinal absorption [18,77]. In conclusion, calcium supplementation in MAs must be used cautiously, and a two-hour interval between oral calcium and antibiotic intake is warranted to minimize potential interactions in MAs.

The use of creatine supplementation is a widespread phenomenon in MAs, as previously reported [62]. Creatine, an amino acid derivate, has been reported to play a major role in intracellular energy shuttling and cellular energy provision and is widely used for sports recovery and performance enhancement [18,79]. Additionally, caffeine, a trimethylxanthine, has been proven to have stimulatory effects that boost performance in athletic populations; therefore, its use is an effective strategy to improve high-intensity endurance exercise and high-intensity efforts, in which individual variation in the ergogenic response must be observed [80]. Nevertheless, some side effects, such as tachycardia or heart palpitations and a potential negative impact on sleep, must be considered [80,81]. Low doses of caffeine (<3 mg/kg body mass, up to 200 mg), in beverages or in alternative forms like tablets/capsules, have been reported to show ergogenic effects in sport and exercise situations: vigilance and alertness appear to be positively influenced by alterations in the nervous system, potentially reduced pain perception, and potential immune system stimulation following exercise [82,83,84,85].

In conclusion, several DSs are widely used by MAs to maintain health conditions and exercise performance. However, the current scientific literature and the available evidence are scarce regarding potential interactions with accompanying medications and DSs in MAs. Further initiatives and research are warranted to establish stronger causal associations and provide MAs with detailed and evidence-based recommendations on DSs and their potential interactions.

## 5. Clinical Implications and Future Directions

Since the number of MAs has steadily increased over the last decade, rigorous medical care that takes into account all the possible concerns and problems of these athletes is urgently needed. At present, there is a high demand for prospective long-term follow-up data that take into account all the uncertainties and limitations of the currently available data [1]. The data collected so far have not sufficiently taken into account relevant aspects of MAs, such as their dietary habits, training schedules, gender-specific differences, and lifestyle habits, and they have not been incorporated into analyses of possible controversial cardiovascular events [1].

AF has a relatively strong known association with extreme endurance training, although a causal relationship has not yet been established [1]. Future long-term observational studies in MAs should focus on identifying specific phenotypes as predictive risk factors for paroxysmal AF, long-term observational medical follow-ups, and new echocardiographic and MRI imaging modalities to verify predictive factors in this growing cohort [1]. The consensus endorsed by experts in the field of ECG monitoring using wearables indicates that heart rate monitors are a reliable tool for training control and arrhythmogenesis detection, which should demonstrate the importance of this topic for the future of medical care in ambitious endurance sports [1,86].

Previous data revealed that male endurance MAs are at increased risk for the development of CAC compared with sedentary low-risk controls [1,3,34,35,36]. Currently, outcome data do not indicate a negative impact of high-intensity and high-volume endurance training on CAC-associated cardiovascular mortality [1,37]. Further scientific efforts are urgently needed regarding the two phenomena of accelerated CAC and MF in endurance MAs to elucidate the specific risk constellations and possible specific phenotypes for individual MA risk assessment [1,3,36,37].

In daily clinical practice, sports medicine specialists face the challenge of caring for elite athletes with and without pre-existing cardiovascular disease. Cardiac assessments of athletes in general and MAs in particular can be challenging in daily routines due to the multitude of structural, functional, and electrical physiological changes that overlap with pathological cardiac disorders [87]. Therefore, international guidelines, which traditionally focused on disqualifying and terminating the competitive sports participation of individuals with cardiac disease, have adopted a more generous attitude based on shared decision-making and careful individual risk assessment [87]. The differential diagnosis of athlete’s heart and cardiomyopathy has commonly been confined to male athletes with a large body surface area participating in endurance sports, such as rowing, long-distance cycling, and long-distance running, over periods of several years [88,89]. A structured ECG analysis has been established to screen athletes for high-risk cardiovascular conditions (HRCCs), with ECG criteria specified and recommended for use in young athletes aged <35 years [90,91]. These international criteria for ECG interpretation in athletes—known as the revised Seattle criteria for ECG interpretation—provide a clear guide for ECG interpretation in athletes [90]. The current scientific data on the transferability of these screening criteria to the cohort of MAs suggest its practicability [91]. Nevertheless, an evaluation of the three established ECG criteria sets, i.e., the European Society of Cardiology (ESC)-2005, Seattle, and International ECG criteria sets, showed their potential application to MAs for resting ECG analyses [90,91]. The most common HRCC, which was not sufficiently detected by the Seattle and International ECG criteria, was CAD, with an incidence of 2.1/100,000 athletes per year [35,91,92]. HCM and CAD represent the most common entities for ScD in MA, and careful ECG screening should be performed by sport cardiologists to detect these two entities by observing specific ECG abnormalities, such as pathological Q waves, signs of LV hypertrophy, ST-segment deviation, T wave inversion, or left bundle branch blocking (LBBB) [93,94,95]. Nevertheless, the associated ECG abnormalities must be judged individually, referring to the athlete’s sport-specific and ethnic background. Clearly, resting ECG screening does not represent an ideal screening approach for CAD detection. Therefore, the athlete’s family history and symptoms; a risk assessment including age, smoking, and hypercholesterinemia; and exercise testing should be taken into consideration when judging an individual athlete’s risk of HRCCs [91]. Coronary CT angiography (CCTA) might represent an interesting additional imaging technique to identify CAD in MAs, but up-to-date, dedicated studies in MAs with long-term outcomes are lacking [96,97]. In this context, shared decision-making strategies and informed patient care are particularly important in modern individualized medical care [1,98].

## 6. Conclusions

In conclusion, regular physical activity represents the most effective preventive strategy against cardiovascular morbidity and mortality. The current general guidelines recommend 150 min of moderate anaerobic exercise or 75 min of vigorous-intensity physical activity per week for the general population. Scientific research has revealed that even the high-volume and high-intensity exercise and the conscious lifestyle of MAs contribute to a life expectancy extension of 5–7 years compared to sedentary individuals [99]. EICR associated with lifelong endurance training has been reported in the context of athlete’s heart in several studies; specifically, an elevated risk of AF and a lower risk of ventricular arrhythmogenesis have been revealed [11]. These cardiovascular controversies must be discussed and taken into consideration in the sports medical assessment and care of these endurance MAs. Prior studies have revealed controversial data regarding the effect of detraining on athlete’s heart. These data are challenged by their heterogeneity, where some data reveal beneficial effects in terms of ventricular chamber size reduction with detraining, and some display no effects of detraining, even after decades [11,100,101,102,103]. The risk of life-threating malignant ventricular arrhythmogenesis is overwhelmingly low, as it mostly derives from hereditary or congenital cardiac disorders [13]. Male endurance MAs seem to be at increased risk of early-onset AF episodes due to EICR and a degree of permanent atrial remodeling, even after detraining [104]. In summary, MAs have a higher prevalence of AF as compared to non-athletic controls, regardless of whether the MAs are retired or detrained or continuously active [11]. Sports medicine practitioners should be aware that EICR promotes a global propensity to arrhythmogenesis, and that pro-arrhythmogenic remodeling in endurance MAs might be sustained and be minimally responsive to detraining [11].

Another topic to be addressed in the future is the potential interaction of accompanying medications and supplements in MAs. On this matter, the current scientific literature and available evidence are scarce. Further initiatives and research are warranted to provide detailed and evidence-based recommendations on supplements and their potential interactions in MAs.

The current scientific data predominantly focus on male endurance MAs; therefore, evaluating the gender-specific risk of physical activity and associated cardiovascular controversies will represent an important future research direction. In this context, hormonal influences, as well as sex-specific risk thresholds, must be discussed against the background of data limitations. Data on longevity indicate a longer life for female athletes compared to sedentary counterparts, and no convincing association between extreme exercise and cardiovascular disease in female MAs has been revealed [105]. Female athletes display fewer signs of cardiac adaptive remodeling on ECG, have a lower incidence of SCD, and are less likely to suffer from AF and CAC during their sports careers [3,105]. The underlying mechanisms of these gender-specific differences are not very well understood; therefore, future scientific efforts are warranted to determine the specific mechanisms of cardiac remodeling in female MAs. The mechanisms described in our review mainly focused on male MAs, who have been predominantly analyzed in the previous research.

In summary, profound expertise in sports and exercise cardiology is necessary to provide individualized cardiological care and follow-up for athletes. Future scientific efforts, addressed in large-cohort studies with long-term follow-up, will be necessary to better understand the various influencing pathophysiological components and gender-specific differences in EICR.

## Figures and Tables

**Table 1:** Prevalence of atrial fibrillation in master endurance athletes—selected studies.

Author	Study Type	Sport	Age	Prevalence
D’ Ambrosio et al., 2025 [11]	Cross-sectional analysis of observational studies	Endurance athletes	>40 y	32% AF 9% ns-VT
Liu et al., 2022 [12]	Retrospective single-center study—ablation of AF	Endurance athletes (running, swimming)	n.a.	n.a.
Shapero et al., 2016 [24]	Electronic Internet-based survey	Boston master athletes	>35 y	9% AF and CAD
Johansen et al., 2022 [26]	10-year survey	Long-distance ski racers	>65 y	28.5% AF
Andersen et al., 2013 [28]	Register data	Vasaloppet, 90 km cross-skiing	total	1.5–2.0% arrhythmias

Abbreviations: y, years; AF, atrial fibrillation; ns-VT, non-sustained ventricular tachycardia; n.a., not applicable; CAD, coronary artery disease; km, kilometers.

## Data Availability

Not applicable.
